# Non-Diabetic Hyperglycemia Exacerbates Disease Severity in *Mycobacterium tuberculosis* Infected Guinea Pigs

**DOI:** 10.1371/journal.pone.0046824

**Published:** 2012-10-04

**Authors:** Brendan K. Podell, David F. Ackart, Natalie M. Kirk, Sarah P. Eck, Christopher Bell, Randall J. Basaraba

**Affiliations:** 1 Department of Microbiology, Immunology and Pathology, College of Veterinary Medicine and Biomedical Sciences, Colorado State University, Fort Collins, Colorado, United States of America; 2 Department of Health and Exercise Science, College of Applied Human Sciences, Colorado State University, Fort Collins, Colorado, United States of America; Johns Hopkins University School of Medicine, United States of America

## Abstract

Hyperglycemia, the diagnostic feature of diabetes also occurs in non-diabetics associated with chronic inflammation and systemic insulin resistance. Since the increased risk of active TB in diabetics has been linked to the severity and duration of hyperglycemia, we investigated what effect diet-induced hyperglycemia had on the severity of *Mycobacterium tuberculosis* (Mtb) infection in non-diabetic guinea pigs. Post-prandial hyperglycemia was induced in guinea pigs on normal chow by feeding a 40% sucrose solution daily or water as a carrier control. Sucrose feeding was initiated on the day of aerosol exposure to the H37Rv strain of Mtb and continued for 30 or 60 days of infection. Despite more severe hyperglycemia in sucrose-fed animals on day 30, there was no significant difference in lung bacterial or lesion burden until day 60. However the higher spleen and lymph node bacterial and lesion burden at day 30 indicated earlier and more severe extrapulmonary TB in sucrose-fed animals. In both sucrose- and water-fed animals, serum free fatty acids, important mediators of insulin resistance, were increased by day 30 and remained elevated until day 60 of infection. Hyperglycemia mediated by Mtb infection resulted in accumulation of advanced glycation end products (AGEs) in lung granulomas, which was exacerbated by sucrose feeding. However, tissue and serum AGEs were elevated in both sucrose and water-fed guinea pigs by day 60. These data indicate that Mtb infection alone induces insulin resistance and chronic hyperglycemia, which is exacerbated by sucrose feeding. Moreover, Mtb infection alone resulted in the accumulation tissue and serum AGEs, which are also central to the pathogenesis of diabetes and diabetic complications. The exacerbation of insulin resistance and hyperglycemia by Mtb infection alone may explain why TB is more severe in diabetics with poorly controlled hyperglycemia compared to non-diabetics and patients with properly controlled blood glucose levels.

## Introduction

Historically the risk factors most frequently linked to TB are HIV infection, tobacco use, extremes in age, alcoholism, malnutrition, chronic kidney disease and both type 1 and type 2 diabetes [1]. How these conditions increase the susceptibility to Mtb infection is poorly understood but is generally thought to be associated with altered cellular immune responses. Even though diabetes has been known for centuries to increase the risk of active TB [2], [3] the current diabetes epidemic has the potential to significantly hamper efforts to control the spread of TB especially in parts of the world where Mtb is endemic [4], [5]. It is estimated that 80% of the global diabetic population is concentrated in developing countries with the incidence estimated to increase from 285 million currently to 439 million by the year 2030 [6]. In regions where the incidence of diabetes surpasses HIV the cumulative TB risk associated with diabetes has the potential to equal or exceed that linked to HIV infection despite the lower relative risk of TB in diabetics compared to HIV [1], [7].

The link between diabetes and TB susceptibility has been recently reaffirmed through retrospective review of clinical and observational studies, which show that regardless of study design and population, diabetes increases the risk of active TB [8]. These clinical studies have revealed that diabetics with active TB have more frequent radiographic evidence of multilobar disease [9], an increased incidence of cavitary lesions [10], [11], increased risk of extrapulmonary dissemination [12], [13], increased sputum bacterial counts [14], and more frequent TB drug treatment failures and higher mortality compared to non-diabetics [15], [16].

Recent studies have shown that despite the numerous metabolic derangements associated with type 2 diabetes, chronically elevated blood glucose (hyperglycemia) especially, is linked to the increased risk of active TB. Patients with glycated hemoglobin (HbA1c) levels exceeding 7%, indicating poorly regulated blood glucose, have a higher risk of developing TB than diabetics with properly controlled blood glucose levels [17], [18]. However hyperglycemia, the diagnostic feature of both type-1 and type-2 diabetes, can also occur in non-diabetics and can presumably also increase the risk of active TB. The relationship between non-diabetic hyperglycemia and the susceptibility to Mtb has not been adequately investigated yet is an important consideration since it is estimated that the pre-diabetic population worldwide currently exceeds 280 million people and is rapidly growing [19]. Non-diabetic hyperglycemia as a TB risk factor was prioritized as important and in need of more research by a panel of experts commissioned to develop a research agenda addressing how the growing diabetes epidemic impacts TB treatment responses and thus global TB control measures [20].

In individuals with type 2 diabetes or pre-diabetes, hyperglycemia is due in part to decreased responsiveness of tissues to the hormone insulin, a phenomenon known as insulin resistance. How hyperglycemia or insulin resistance increases the susceptibility to TB whether in diabetics or non-diabetics is unknown [21]. One possibility may be related to the chemical interaction of reducing sugars including glucose, with proteins and other host macromolecules known as the Maillard reaction. The non-enzymatic and therefore unregulated binding of sugar residues to proteins (glycation) is associated with the normal aging process but is exacerbated by chronic elevations in blood glucose in individuals with poorly controlled diabetes. Glycation and the intermediate formation of Amadori products is a reversible reaction, which precedes the formation of irreversibly modified adducts referred to as advanced glycation end products (AGEs) [22]. The accelerated accumulation of AGEs in diabetic patients is implicated in the pro-inflammatory responses directly linking hyperglycemia to the debilitating and life-threatening complications of diabetes including nephropathy, neuropathy, retinopathy and atherosclerosis [23], [24], [25].

Because diabetes is an important TB risk factor and little is known about how hyperglycemia alone increases the risk of Mtb infection, we investigated the impact diet-induced hyperglycemia had on the severity of experimental Mtb infection and AGE accumulation in non-diabetic guinea pigs. The design of this study was based on the hypothesis that the consequences of hyperglycemia and TB share a common pathogenesis involving chronic inflammation and the formation of AGEs and that combining the two conditions exacerbates TB severity even in hyperglycemic, non-diabetic guinea pigs.

## Materials and Methods

### Ethics Statement

All experimental protocols were in accordance with the National Research Council's Guide for the Care and Use of Laboratory Animals and were approved by the Animal Care and Usage Committee at Colorado State University under protocol number 09-140A-03.

### Animal Treatments

A total of 60 guinea pigs (Charles River Laboratories; North Wilmington, MA) were randomly assigned to 4 treatment groups split between two separate experiments: Mtb infected and sucrose-fed (n = 20), Mtb infected and water-fed (n = 20), uninfected and sucrose-fed (sucrose control, n = 10) and uninfected and water-fed (uninfected control, n = 10). All animals treated with sucrose were given 400 mg of sucrose as a 40% w/v solution *per os* daily beginning on the day of infection based on an unpublished observation that this dose of sucrose increases the severity of TB in guinea pigs when used as a carrier control in anti-TB drug treatment studies. In addition, a slightly lower dose of sucrose induces post-prandial hyperglycemia and insulin resistance in the rat [26]. This 400 mg dose effectively increases post-prandial blood glucose levels with a peak average of 2.14 fold above baseline in the guinea pig (Figure S1). The mock-treated, water-fed animals were given an equivalent volume of water *per os* daily also beginning on the day of infection.

### Aerosol Infection of Guinea Pigs with *M. tuberculosis*

Culture stocks of *Mycobacterium tuberculosis* strain H37Rv (TMC #102, Trudeau Institute; Saranac Lake, NY) collected at mid-log phase of growth in Proskauer-Beck liquid medium containing 0.05% Tween-80 were diluted to 1×10^6^ CFU/ml and delivered by low-dose aerosol infection using a Madison chamber aerosol generation device calibrated to deliver approximately 20 bacilli to each animal.

### Euthanasia and Sample Collection

At days 30 and 60 of infection, 10 guinea pigs from each infected treatment group and 5 guinea pigs from each uninfected group were anesthetized by intramuscular injection of ketamine (20 mg) and diazepam (1 mg) prior to humane euthanasia with an overdose of sodium pentobarbital by intraperitoneal injection (1.5 ml/kg). Lung, spleen and mediastinal lymph node was collected either for histopathology and fixed in 4% paraformaldehyde or for Mycobacterial culture and weighed prior to homogenization and plating of serial dilutions. All paraformaldehyde fixed tissue was removed after 72 hours and placed in 70% ethanol for storage.

### Tissue CFU Quantification

Bacterial burden in the lung, spleen, and mediastinal lymph nodes was determined by plating serial dilutions of tissue homogenates on nutrient 7H11 agar followed by counting colony-forming units after incubation at 37°C for 3–6 weeks. Data was expressed as CFUs per gram of tissue.

### Histopathology and Lesion Analysis

Either on day 30 or day 60 of infection, lung, spleen, and mediastinal lymph node were collected and fixed in buffered 4% paraformaldehyde for 3 days then stored permanently in 70% ethanol. Tissues were paraffin embedded, sectioned at 5 µm and stained with hematoxylin and eosin by standard methods.

Total lung, lymph node and spleen tissue area for each organ was quantified using the Stereo Investigator software version 10.02 (MBF Bioscience; Williston, VT) and the Nikon Eclipse 80i microscope. Total lesion area was compared to total tissue area of lung, lymph node or spleen respectively to yield total lesion burden expressed as a percentage of lesion to total tissue area using the area fraction fractionator as previously described [27]. Similarly, area ratio quantification was performed comparing necrosis to lesion area.

### Serum Glucose and Oral Glucose Tolerance Test

Serum was collected from guinea pigs at the time of euthanasia on either day 30 or day 60 of infection. Glucose was measured using the glucose oxidase enzymatic method (Cayman Chemical; Ann Arbor, MI) at an absorbance of 500 nm on a microplate spectrophotometer. The oral glucose tolerance test was performed on the animals after 12 hours of overnight fasting. At time 0 each guinea pig was administered an oral dose of D-glucose (Sigma-Aldrich; St. Louis, MO) of 1 g/kg. Percutaneous whole blood obtained from an ear pinna prick site was used for sequential glucose measurements performed at 0, 60, and 120 minutes post-glucose administration with a handheld glucometer (Freestyle Lite, Abbott Diabetes Care; Alameda, CA) validated against the glucose oxidase assay.

### Immunohistochemistry for AGEs

Immunohistochemistry was performed on paraformaldehyde-fixed, paraffin-embedded 5 µm sections of lung from the sucrose-fed and water-fed guinea pigs targeting AGEs. Slides containing tissue sections were deparaffinized and rehydrated followed by antigen retrieval (PT Module Buffer 4, Thermo Scientific; Rockford, IL) at an incubation temperature of 95°C for 30 minutes. Endogenous peroxidase activity was quenched with 0.3% hydrogen peroxide treatment for 10 min, rinsed in Tris-buffered saline with 1% Tween-20 (TTBS), and followed by two blocking steps: (i) 15 min incubation with 0.15 mM glycine in PBS, and (ii) 30 min incubation with 10% fetal bovine serum and 1% bovine serum albumin. The slides were then incubated for 30 minutes with rabbit polyclonal antibody to AGE (ab23722, AbCam; Cambridge, MA) or rabbit polyclonal IgG (ab27478, AbCam; Cambridge, MA) at a 1∶400 or 1∶100 dilution, respectively, in blocking buffer. After rinsing, the slides were incubated for 30 minutes with biotinylated goat-anti rabbit IgG antibody (Vector Laboratories; Burlingame, CA). Horseradish peroxidase was added followed by diaminobenzidine substrate to visualize bound antibody. Hematoxylin was used as a counterstain.

Evaluation of immunohistochemical reactivity within TB lesions of lung tissue was based on a 4-point scoring system and performed by a pathologist blinded to the study groups. Areas of lung containing primary lesions with or without necrosis were separated from lesion-free lung and scored based on the following 2 criteria: (i) Percent of lesion inflammatory cells displaying immunoreactivity: 0- No immunoreactivity is evident, 1- Up to 25% of cells are reactive, 2- Up to 50% of cells are reactive, 3- Up to 75% of cells are reactive, 4- Greater than 75% of cells are reactive. (ii) Intensity of immunoreactivity was assessed as follows: 0- none, 1- mild, 2- moderate, 3- severe. The maximum possible score for AGE IHC is 7.

The specificity of anti-AGE antibodies for AGE-modified guinea pig proteins was validated by competitive inhibition of tissue binding utilizing AGE-modified bovine serum albumin (ab51995, Abcam; Cambridge, MA) at an antibody:antigen ratio of 1∶100 prior to adding the primary antibody to each tissue section. Specificity was confirmed by loss or lack of immunoreactivity in tissue sections indicating effective blocking of anti-AGE antibodies to guinea pig lung tissue. Purified polyclonal rabbit IgG was applied as a primary antibody for a negative control.

### Quantification of Serum AGE Levels

Protein content of serum was measured using the BCA assay (Thermo Scientific; Rockford, IL) and samples were diluted to a concentration of 10 µg/ml. AGE levels were measured in diluted serum samples by ELISA (Cell Biolabs; San Diego, CA). Serum samples were analyzed on a standard curve of AGE-modified bovine serum albumin as directed by the manufacturer and expressed as the mass of AGE-modified serum protein per 10 µg analyzed. This assay detects AGE structures formed on proteins in the presence of glycolaldehyde including two of the most prevalent, carboxymethyllysine and pentosidine.

### Quantification of Serum Free Fatty Acid Levels

Serum free fatty acid levels were measured by fluorescence in an assay utilizing a coupled enzymatic reaction (Cayman Chemical; Ann Arbor MI). Briefly, acyl CoA synthetase catalyzes fatty acid acylation of coenzyme A. The acyl CoA generated is oxidized by acyl CoA oxidase to generate hydrogen peroxide, which in the presence of horseradish peroxidase and 10-aceyl-3,7-dihydroxyphenoxazine (ADHP) generates fluorescence that is measured spectrophotometrically at an excitation wavelength of 530 nm and an emission wavelength of 585 nm.

### Data Analysis

Statistical analyses and graphic expression of the data was by the use of the statistical package in GraphPad Prism 5. Bacterial tissue burden was log_10_ transformed and normalized to per gram of tissue prior to analysis to ensure approximate normal distribution with a common variance. Comparison of paired observations based on a single treatment was performed by paired t test. The differences between treatment groups of the remaining data was compared using a two-way ANOVA followed by Bonferroni post-test for pair-wise comparison of means with significance set at P≤0.05.

## Results

### Serum Glucose and Glucose Tolerance Tests

**Figure 1:**
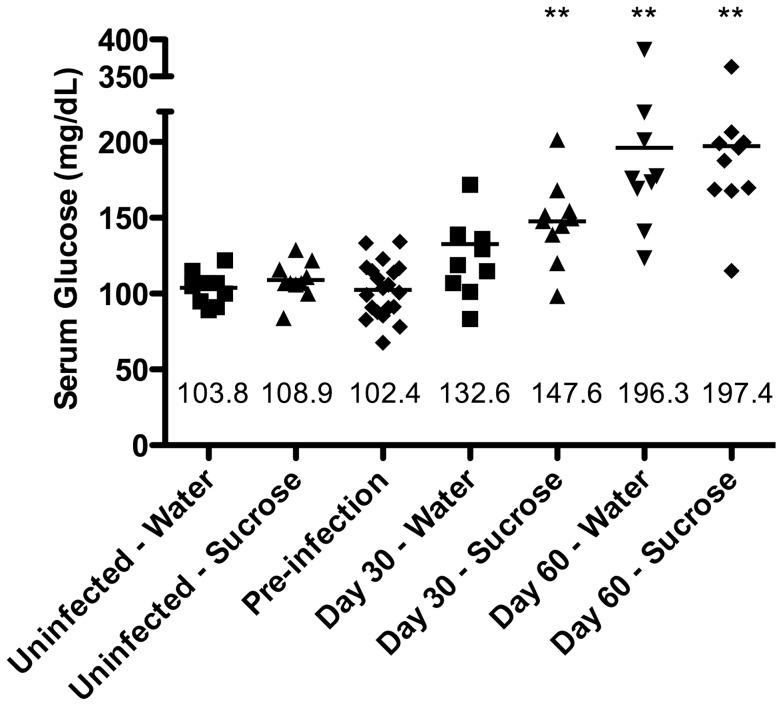
Hyperglycemia resulting from Mtb infection was exacerbated early by sucrose treatment. Random sampling of serum glucose values were compared to the mean glucose level of guinea pigs prior to sucrose feeding or infection (Pre-infection, n = 20) and served as the normal reference value. Serum glucose values of uninfected guinea pigs are similar to normal pre-infection values. Despite a mild increase in serum glucose associated with Mtb infection in the water-fed controls, significant exacerbation of hyperglycemia was only induced in sucrose-fed guinea pigs on day 30 of infection (n = 10). However,this difference was independent of sucrose feeding by day 60 (n = 9) of infection since persistent hyperglycemia was present in both sucrose- and water-fed groups. **p≤0.01.

At day 30 of infection, serum glucose levels (Figure 1) were elevated with a mean value of 132.6 mg/dl in infected water-fed control guinea pigs but this increase was not statistically significant (p>0.05). In contrast, hyperglycemia was further exacerbated by sucrose feeding at 30 days of infection with a mean value of 147.6 mg/dl (p≤0.01). Interestingly, similar hyperglycemia was evident by 60 days of infection in both the water- and sucrose-fed groups with mean values of 196.3 and 197.4 mg/dl, respectively (p≤0.01).

Oral glucose tolerance tests (OGTT) were performed prior to euthanasia at both days 30 (Figure S2A) and 60 (Figure S2B) of infection to confirm that sucrose feeding or infection alone or in combination did not induce a diabetic type response to oral glucose challenge. A response similar to that of normal water-fed controls was present in all groups independent of sucrose feeding or infection with a peak glucose concentration of approximately 2 fold at 60 minutes and a return to <1.5 fold by 120 minutes post-challenge.

### Histopathology

**Figure 2:**
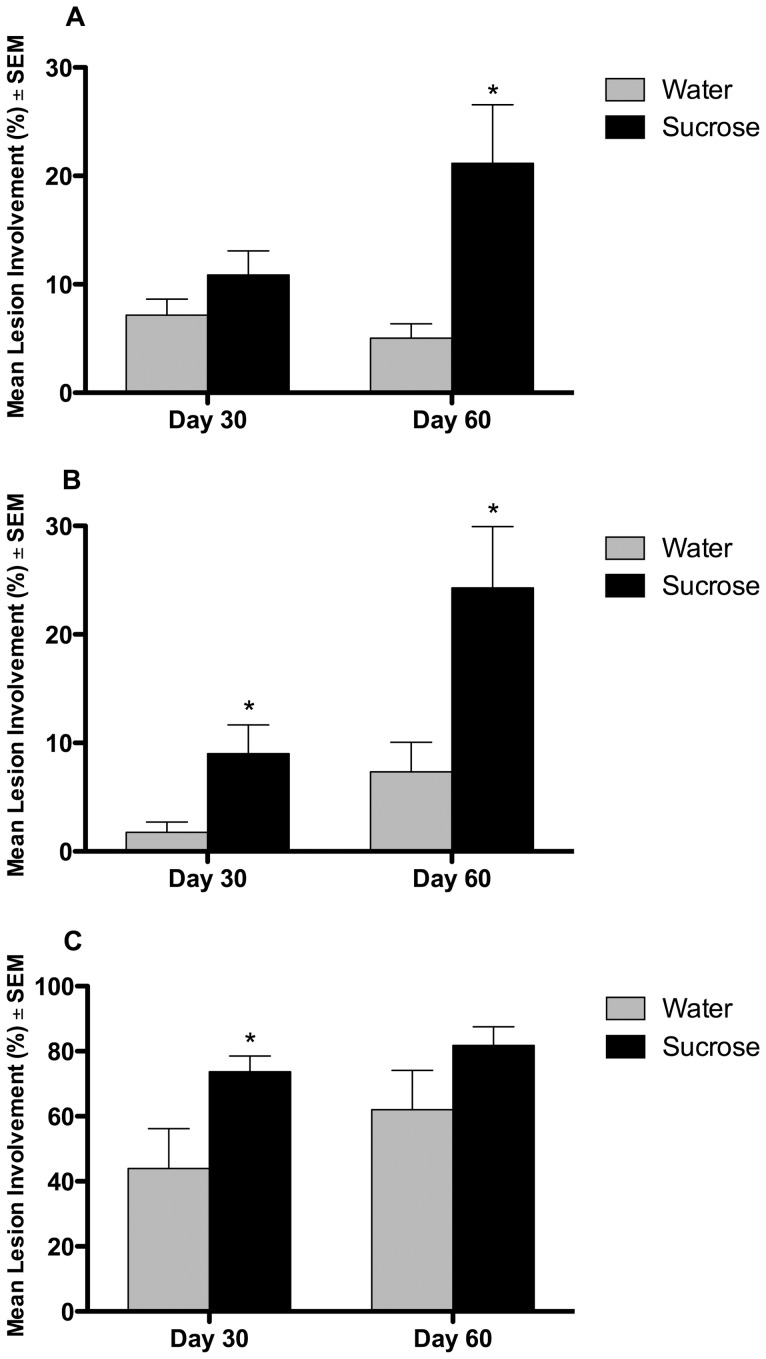
Sucrose fed guinea pigs had significantly higher lung and extrapulmonary Mtb lesion burden. The lung and lesion area was determined from hematoxylin and eosin stained tissue sections for each animal and the data expressed as mean percent involvement for each treatment group. The sucrose-fed guinea pigs had a significantly higher lesion burden compared to the water-fed control group in the lung (A) on day 60 of infection, spleen (B) on days 30 and 60 of infection, and mediastinal lymph node (C) on day 30 of infection. n = 10, *p≤0.05.

Pulmonary pathology showed no statistically significant quantitative differences between the sucrose-fed animals compared to water-fed controls on day 30 of infection (p>0.05) but was more severe in the sucrose-fed group by day 60 (p≤0.05) (Figure 2A). More striking was the increase in size and frequency of the extrapulmonary lesions in the spleen (Figure 2B) and lymph node (Figure 2C) of sucrose-fed guinea pigs compared to the water-fed controls. In the spleen, the lesion burden was significantly higher in sucrose-fed guinea pigs by day 30 of infection (p≤0.05) with an even greater difference in severity by day 60 (p≤0.05). At day 30 of infection, the lesion burden in mediastinal lymph nodes was significantly higher in the sucrose-fed group (p≤0.05), which progressed in both groups such that there were no differences between water and sucrose-fed groups by day 60 of infection (Figure 2C). The degree of necrosis, measured as percent necrosis area to lesion area, was not significantly different between water and sucrose-treatment in any organ at either day 30 or day 60 of infection (p≤0.05) (Figure S3).

**Figure 3:**
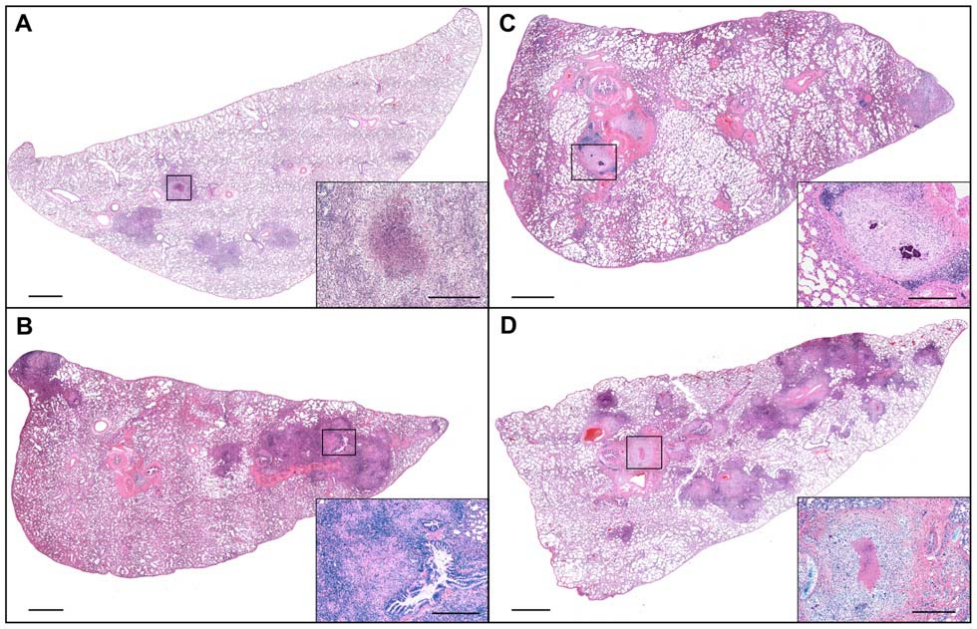
Sucrose feeding resulted in a more severe pulmonary lesion burden in *Mtb* infected guinea pigs. Images represent the animals closest to the mean values for severity of lesion burden as determined by measuring lesion and normal lung area using morphometric analysis (see Figure 2). Pulmonary lesion severity was similar between water- (A) and sucrose-fed (B) guinea pigs at day 30 of infection but was more severe in the sucrose-fed animals by 60 days of infection (D) compared to the water-fed controls (C). Bar = 1000 µm. Hematoxylin and eosin stain. *Insets*: High magnification views of the TB lesions delineated on the subgross views of A–D; Bar = 100 µm.

**Figure 4:**
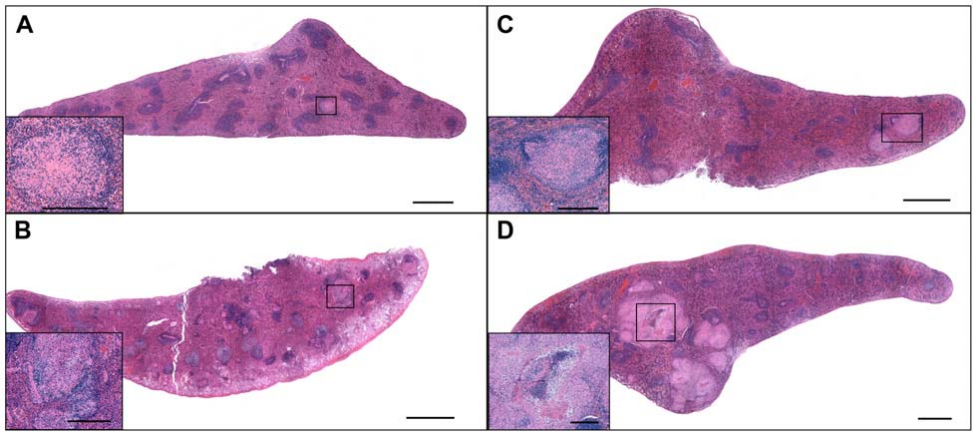
Spleen lesions from extrapulmonary dissemination of Mtb were more severe and more numerous in sucrose-treated guinea pigs. Images represent the animals closest to the mean severity of lesion burden as determined by measuring lesion and normal spleen area using morphometric analysis (see Figure 2). Splenic involvement was mild in the sucrose-fed guinea pigs at 30 days of infection (A) which was even less in the water-fed controls (B). However, splenic lesions were significantly more severe in the sucrose-fed animals by day 60 of infection (D) compared to the water-fed control group (C). Bar = 1000 µm. Hematoxylin and eosin stain. *Insets*: High magnification views of the TB lesions delineated on the subgross views of A–D; Bar = 100 µm.

The qualitative differences of lesions for each treatment group are depicted in representative photomicrographs of lung in Figure 3 and spleen in Figure 4. Individual lesions were represented by well-delineated granulomas consisting of epithelioid macrophages and scattered multinucleated giant cells with fewer lymphocytes in sections of lung, lymph node and spleen of both treatment groups by day 30 of infection with necrosis and central accumulations of infiltrating granulocytes. By day 60 of infection the granulomas had expanded in size, as did the extent of central lesion necrosis, peripheral lymphocytic infiltration, circumferential fibrosis and central dystrophic mineralization. Non-necrotic secondary pulmonary lesions indicative of hematogenous reinfection of the lung in sucrose-fed animals were more pronounced by day 30 of infection and progressed in size and number by day 60 of infection. Based on histologic examination the cellular constituents were similar between treatment groups however the size and frequency of lesions were more severe in the sucrose-fed groups.

### Tissue Bacterial Burden

**Figure 5:**
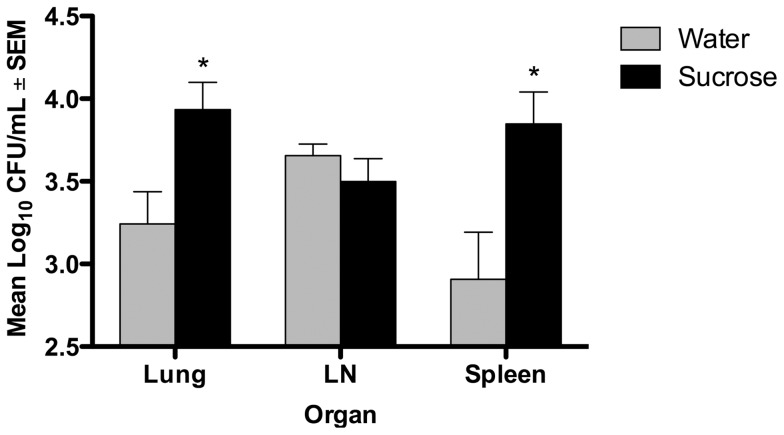
Sucrose treatment resulted in higher bacterial burden in lung and spleen of *Mtb* infected guinea pigs. Yield of viable bacilli from the lung was significantly higher in sucrose-fed guinea pigs on day 60 of infection compared to water-fed control animals. Even higher yields of bacilli were recovered from the spleens of sucrose-fed guinea pigs on day 60 of infection. No significant differences were observed in the mediastinal lymph nodes (LN). n = 10, *p≤0.05.

On day 30 of infection similar numbers of viable bacilli were cultured from the lungs of water- and sucrose-fed groups (p>0.05, data not shown). There was a significant increase in the lung and spleen bacterial burden by day 60 of infection (p≤0.05) (Figure 5). Bacterial numbers in the mediastinal lymph node on day 60 of infection were not statistically different between sucrose- and water-fed, infected groups (p>0.05). While the difference in bacterial numbers in the lung of sucrose-fed animals was significant, the differences between treatment groups as reflected by spleen bacterial numbers were even greater.

### Serum Free Fatty Acid Levels

**Figure 6:**
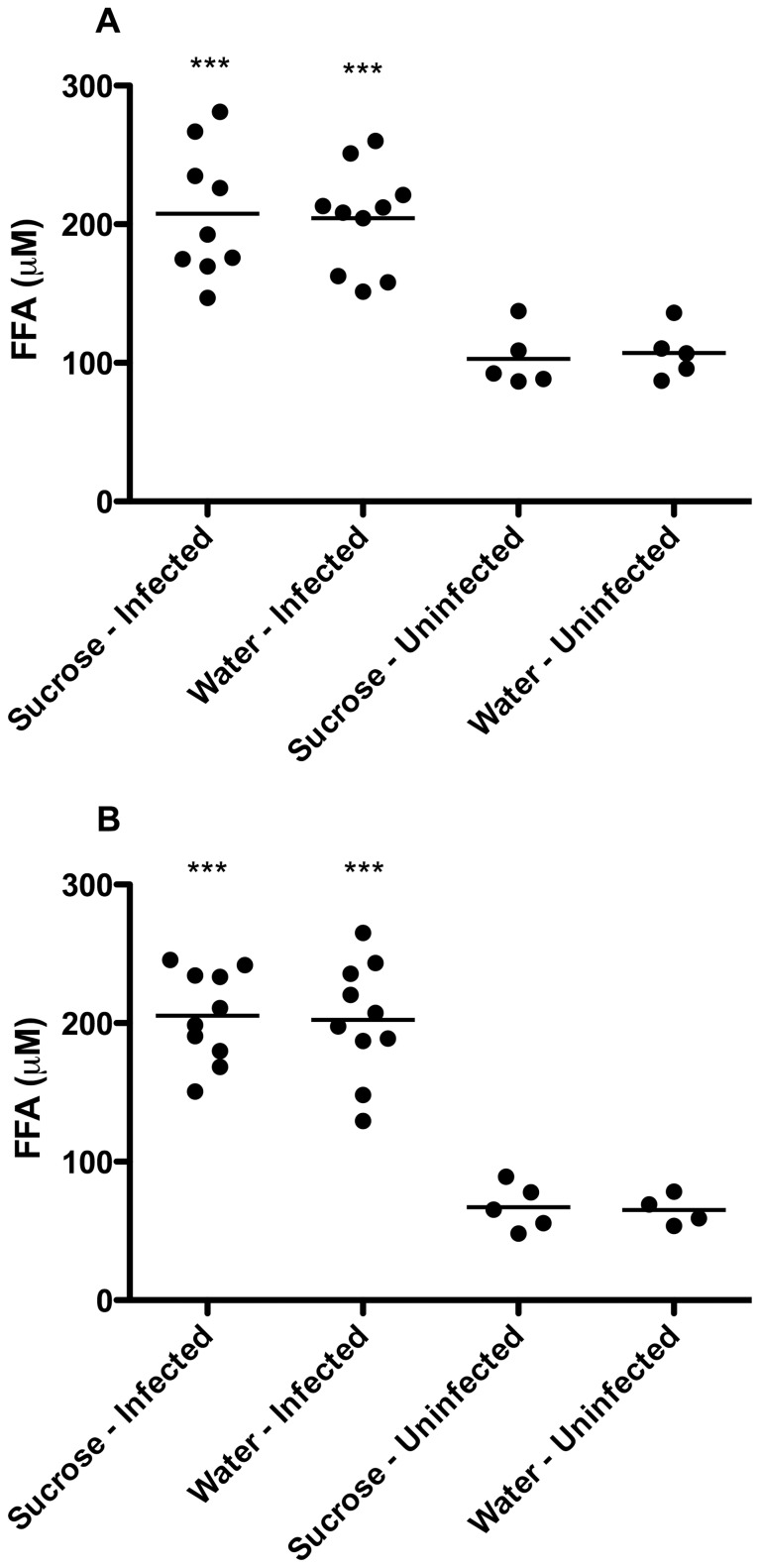
Elevated total serum free fatty acids occurred as a result of Mtb infection and not due to sucrose treatment. Serum free fatty acids are similarly elevated between sucrose- (n = 9) and water-fed (n = 10) Mtb infected guinea pigs at day 30 of infection (A). The levels remain similarly elevated in sucrose- (n = 10) and water-fed (n = 10) Mtb infected guinea pigs at day 60 of infection with no increase in the mean values over time (B). Serum free fatty acid levels in sucrose-fed uninfected animals (day 30, n = 5; day 60, n = 5) are comparable to water-fed controls (day 30, n = 5; day 60, n = 4) at both time points. ***p≤0.001.

Total free fatty acids were measured in serum from guinea pigs of all treatment groups at days 30 and 60 of infection (Figure 6). Elevated mean FFA levels were present in both water-and sucrose-fed, Mtb infected groups at both days 30 and 60 of infection (p≤0.001). Compared to mean values of 102.8 µM and 107.2 µM at day 30 of infection in uninfected sucrose- and water-fed guinea pigs, respectively, FFAs were elevated with mean values of 207.6 µM and 204.3 µM in the infected groups. Serum FFAs remained elevated at day 60 of infection with mean values of 205.4 µM and 202.3 µM in sucrose- and water-fed, infected guinea pigs, respectively while uninfected mean values were minimally reduced at 67.2 µM and 65.03 µM. Sucrose feeding alone in uninfected guinea pigs did not result in alterations of serum FFAs compared to water-fed controls (p>0.05).

### Serum AGE Levels

**Figure 7:**
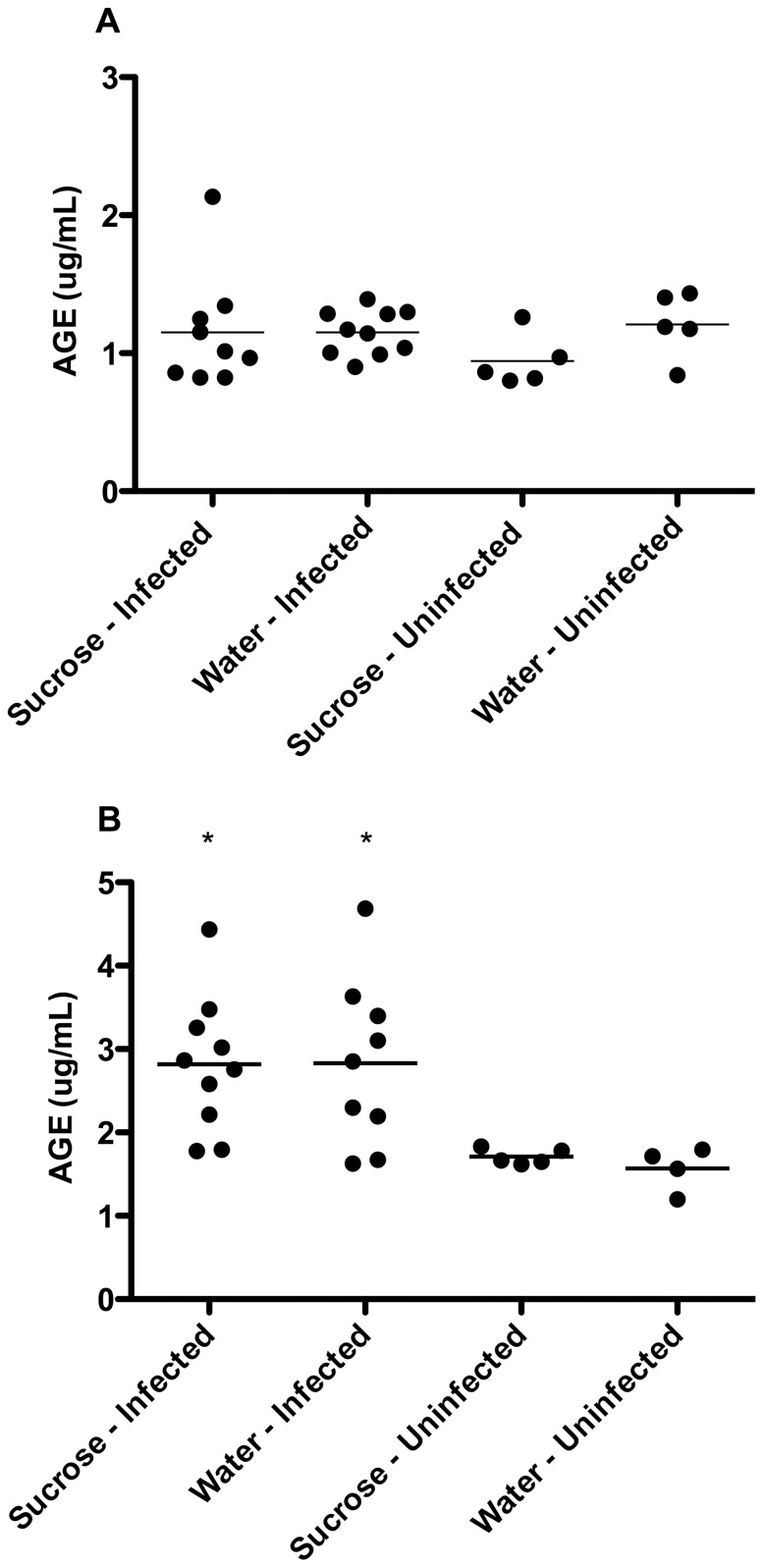
Mtb infection induces the formation and accumulation of AGEs in serum from guinea pigs independent of sucrose feeding. Levels of serum AGEs were analyzed in sucrose- or water-fed guinea pigs with and without Mtb infection at both 30 and 60 days of infection. At 30 days of infection (A), mean serum AGEs are not elevated by either sucrose feeding or Mtb infection with levels remaining similar to uninfected controls. However, elevations in serum AGEs were present at 60 days due to Mtb infection (B) (sucrose-infected n = 10, water-infected n = 9) and were not exacerbated by sucrose feeding. AGE levels in uninfected, sucrose- and water-fed controls (n = 5 and n = 4, respectively) remain similar to levels at day 30. *p≤0.05.

The differences in serum AGEs between treatment groups at days 30 and 60 of infection are illustrated in Figure 7. Elevated serum AGEs were present in a single guinea pig in the sucrose-fed, infected group at day 30 of infection with no reflective increase in mean serum AGEs for that group (p>0.05). All remaining guinea pigs of sucrose- and water-fed, infected groups were similar to uninfected, water-fed controls. In contrast, by day 60 serum AGEs were similarly elevated in both the water- and sucrose-fed infected groups compared to the water-fed uninfected control group (p≤0.05). There was no evidence of increased AGEs due to sucrose feeding alone when compared to water-fed, uninfected controls (p>0.05).

### AGE Immunohistochemistry

Immunoreactivity for AGEs was seen specifically in the pulmonary TB lesions, within the serum and lymph fluid of all blood and lymphatic vessels and associated with extracellular matrix surrounding airways and blood vessels. There was no immunoreactivity seen in other normal microanatomic structures of the lung parenchyma. In both sucrose- and water-fed, uninfected animals, immunoreactivity was limited to intravascular serum, lymph fluid and extracellular matrix and were not significantly different. Immunoreactivity specifically colocalized with cells of the pulmonary TB lesions and most strikingly within the areas of central necrosis of the primary lesions. Occasionally, AGE-positive macrophages were in the lumen exudate, which frequently accumulates within bronchioles in the chronic stages of Mtb infection.

**Figure 8:**
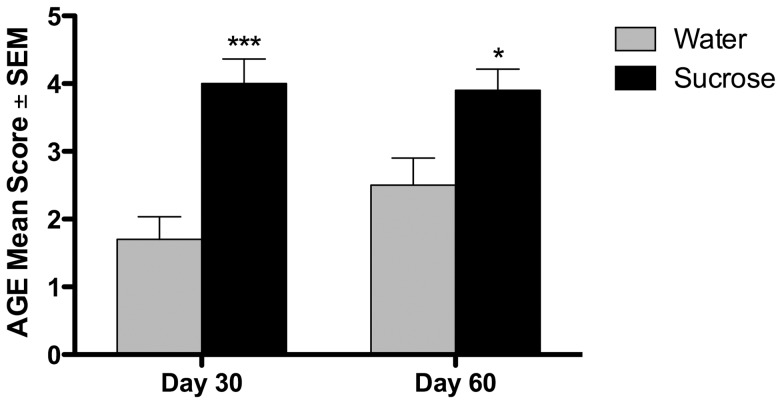
Sucrose feeding of Mtb infected guinea pigs significantly increased tissue AGEs by day 30 of infection. The mean score of AGE immunohistochemistry on lung is depicted in water- and sucrose-fed guinea pigs on days 30 and 60 of infection. Significantly increased AGE formation and accumulation was present within TB lesions by day 30 of infection in the sucrose-fed group compared to the water-fed controls. AGE levels at day 30 were similar to day 60 in the sucrose-fed group but additional AGEs accumulated within lesions by day 60 of the water-fed controls, which was not statistically significant. n = 10, *p≤0.05,***p≤0.001.

**Figure 9:**
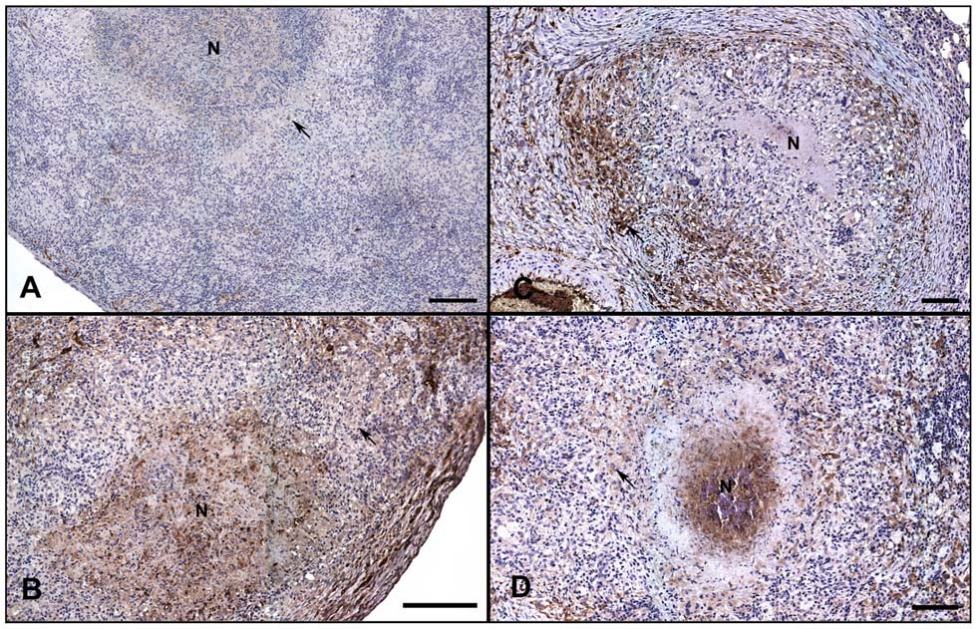
Sucrose feeding of *Mtb* infected guinea pigs increased lesion associated AGEs on day 30 and 60 of infection. AGEs are evaluated by immunohistochemistry on lung tissue sections of *Mtb* infected guinea pigs. The majority of strong immunoreactivity was associated with TB lesions. Immunoreactivity was evident within the cytoplasm of macrophages forming granulomatous lesions and was also strongly present within central necrosis (N) of primary TB pulmonary lesions. (A) Minimal reactivity was confined to the serum, rare macrophages in the lesions (arrow) and minimally within areas of necrosis (N) of the water-fed control group at 30 days of infection. (B) In contrast, at 30 days of infection, the sucrose-fed animals had strong immunoreactivity in the majority of lesion macrophages (arrow) and in areas of necrosis (N). (C) By day 60 of infection, AGEs began to accumulate within macrophages (arrow) in the water-fed controls but minimal reactivity was present in areas of necrosis (N). (D) The sucrose-fed animals had strong immunoreactivity within the majority of macrophages (arrow) and in areas of necrosis (N) at day 60 of infection. All tissue sections depicted are representative of the mean AGE IHC score for each treatment group at each time point (see Figure 8). Bar = 100 µm.

The number and immunostaining intensity of macrophages within lesions was significantly different between treatment groups (Figure 8). The sucrose-fed guinea pigs had a marked accumulation of AGEs within lesions on both days 30 and 60 of infection compared to water-fed guinea pigs (p≤0.001 and p≤0.05, respectively) with elevated but little difference in the sucrose-fed group between days 30 and 60. Interestingly, pulmonary AGEs increased in the water-fed group between days 30 and 60 of infection but did not increase to a level comparable to that of the sucrose-fed animals. However, this increase in the water-fed group was not statistically significant (p>0.05). The differences in IHC scores were due to morphologic differences in macrophage or necrosis localization, frequency of immunoreactivity, and signal intensity in lungs between sucrose- and water-fed guinea pigs (Figure 9). Lungs from sucrose-fed guinea pigs had greater numbers of inflammatory cells with more intense immunoreactivity compared to water-fed guinea pigs. Additionally, there was increased intensity of immunoreactivity present within areas of lesion necrosis in the sucrose-fed compared to water-fed guinea pigs.

## Discussion

In this study we show that daily feeding of a relatively small amount of sucrose in non-diabetic guinea pigs exacerbated Mtb infection-associated hyperglycemia, which significantly increased TB disease severity. Most notable was not only increased lung lesion and bacterial burden but also earlier hematogenous dissemination of bacilli in sucrose-fed animals, which resulted in earlier and more severe extrapulmonary TB. These studies were initiated following a fortuitous observation showing that the use of sucrose as an oral anti-TB drug carrier in guinea pigs resulted in more severe disease compared to non-sucrose fed controls. The importance of these data is that inducing post-prandial hyperglycemia in guinea pigs by supplementing a normal chow diet with sucrose had a significant negative impact on the severity of experimental Mtb infection. These data are significant since it has been shown that hyperglycemia is a TB risk factor and diabetics with uncontrolled hyperglycemia are at greater risk for active TB compared to diabetic patients who have successfully maintained near normal blood glucose levels [17], [18]. What remains unknown however, is whether hyperglycemia in non-diabetic humans also increases the risk and severity of TB as suggested by these data.

Invariably preceding the development of overt type 2 diabetes, there is a decrease in systemic insulin sensitivity referred to as insulin resistance, which is also a feature of chronic inflammatory conditions in non-diabetics. Our results show that hyperglycemia in Mtb infected guinea pigs fed sucrose was largely driven by Mtb infection associated inflammation and sucrose feeding contributed to hyperglycemia in the early stages of infection. Therefore, these data suggest that the persistent hyperglycemia was more likely due to insulin resistance associated with chronic Mtb infection. However, sucrose feeding lengthened the duration and severity of hyperglycemia as was evident by increased clinical disease severity and the formation and accumulation of tissue and serum AGEs.

One possible explanation for how chronic Mtb infection increases blood glucose levels is through the elevation of pro-inflammatory cytokines, specifically TNF-α. TNF-α rapidly induces insulin resistance by stimulating free fatty acid (FFA) synthesis and secretion, which interferes with post insulin receptor signaling via altered phosphorylation of insulin receptor substrates [28], [29], [30]. Our data are consistent with FFA mediated insulin resistance in Mtb infected guinea pigs since FFA levels were elevated in both the water- and sucrose-fed animals, with similar elevations present at both days 30 and 60 of infection.

Although significant glucose intolerance was not demonstrated by OGTT, significant post-prandial hyperglycemia was confirmed 60 minutes after administration of 1 g/kg of glucose administered during the test. However, even a short interval of post-prandial hyperglycemia is known to result in significant oxidative stress in humans [31]. Additionally, oral supplementation of sucrose has been shown to model insulin-resistance in rats as early as 2 weeks after initiation of treatment while persistent hyperglycemia does not manifest until 9 weeks after treatment [26]. In this study, a comparatively low dose of 400 mg of sucrose daily was utilized to mimic the original observation made when sucrose was used as an anti-TB drug carrier. Compared to water-fed controls, this treatment induced more severe hyperglycemia by 30 days when combined with Mtb infection. We showed, however, that combining sucrose feeding and Mtb infection did not induce diabetes since oral glucose tolerance tests did not show any evidence of elevated fasting glucose or glucose intolerance typical of type 2 diabetes.

The degree of hyperglycemia, in particular non-diabetic hyperglycemia, has not been previously investigated as a risk factor for TB in humans and experimentally in animal models. In this study, non-diabetic guinea pigs with hyperglycemia had more severe and earlier onset of extrapulmonary TB as evident by higher lesion burden in the mediastinal lymph nodes at 30 days of infection followed by an increased bacterial burden and increased severity of spleen lesions on both days 30 and 60 of infection. Immunosuppression has been implicated as the mechanism by which diabetes and other TB risk factors increase the susceptibility to Mtb infection or exacerbate active TB disease [1]. While suppression of the adaptive immune response potentially explains the increased numbers of viable bacilli isolated from both lung and spleen of sucrose-fed animals on day 60 of infection there are alternative explanations. The delay in the adaptive immune response as demonstrated in diabetic mouse models of Mtb infection combined with a pro-inflammatory innate response associated with hyperglycemia could also explain our findings [32]. In addition, chronic hyperglycemia and free fatty acids may represent a readily available carbon source for rapidly replicating bacilli [33].

The extrapulmonary spread of bacilli to the regional lymph nodes is a consistent feature of the experimental Mtb infections in animal models including the guinea pig [34]. The rate of dissemination and severity of extrapulmonary lesions is an indicator of increased susceptibility of the host or increased virulence of the challenge strain of Mtb [35], [36]. Studies performed in our laboratory and others have demonstrated an increased resistance to extrapulmonary TB in guinea pigs vaccinated with BCG prior to challenge, but few studies have demonstrated a decreased resistance in this model [34], [36]. Disseminated extrapulmonary TB in humans is associated with HIV infection and the risk increases proportionally with severity of immunosuppression [37], [38]. Similarly, diabetes, a condition known to alter immune function, is associated with increased risk of developing extrapulmonary dissemination of Mtb in humans [12], [13]. In this study, we show that non-diabetic hyperglycemia had a significant negative impact on disease progression resulting in earlier and more severe dissemination of bacilli. A potential mechanism leading to more extensive Mtb dissemination may be related to microvascular damage, a direct consequence of hyperglycemia, which is central to the pathogenesis of diabetic complications [39].

A potential link tying together high carbohydrate diet and a maladaptive pro-inflammatory state is the increased formation of circulating and tissue-associated AGEs. The presence of increased AGEs is tied to both a pro-oxidative and pro-inflammatory state where oxidative stress mediates the persistent inflammatory response [40], [41]. In patients with poorly controlled diabetes and increased glycated hemoglobin (HbA1c), peripheral blood leukocytes express increased innate and type 1 cytokines indicative of a pro-inflammatory phenotype [42], [43]. In this study, sucrose-fed guinea pigs developed more severe pulmonary and splenic inflammation, which corresponded to increased AGE formation in the TB lesions. The specific localization and accumulation of AGEs in TB lesions even in water-fed controls implicates AGEs as a potential driving force for the pro-inflammatory state in TB as well, which is exacerbated by diabetic or non-diabetic hyperglycemia. In part, this may be due to increased oxidative stress, which we have shown to be important in the pathogenesis of TB. Interestingly, this pathogenic mechanism is further supported by our findings that treatment of Mtb infected guinea pigs with the antioxidant drug N-acetyl cysteine significantly reduces disease burden including the extent of extrapulmonary dissemination [44].

There are multiple potential mechanisms for AGE formation in this study. Hyperglycemia may induce AGEs via direct interactions between glucose and serum or extracellular matrix proteins. Alternatively, increased intracellular glucose under hyperglycemic conditions promotes rapid formation of dicarbonyl intermediates of glycolysis including glyoxal, 3-deoxyglucasone and methylglyoxal, all potent inducers of AGE formation [45], [46], [47]. Accumulation of AGEs associated with chronic inflammation has been shown to occur [48] and methylglyoxal [49], as well as its precursor dihydroxyacetone [50], have been identified in association with TB lesions. This demonstrates the potential for at least localized AGE formation even in euglycemic animals infected with Mtb. AGE formation associated with Mtb infection was a prominent feature in this study where AGE accumulation increased in TB lesions between day 30 and 60 of infection even in water-fed guinea pigs. Additionally, elevated serum AGEs at day 60 of infection were equally elevated in both sucrose-fed and water-fed guinea pigs. Recently AGE formation and secretion by bacteria has been shown *in vitro* and may represent a third source of AGEs in Mtb infection [51]. However, the significant increase in tissue AGEs, which resulted from sucrose feeding, suggests that while bacteria and local tissue metabolism may be a source for AGEs during Mtb infection, the accumulation is greatly exacerbated by hyperglycemia.

The importance of understanding the impact diabetes and hyperglycemia have on TB pathogenesis and treatment has been highlighted as a global priority [7]. Aside from overt clinical diabetes, the results herein indicate the potential for hyperglycemia in a non-diabetic state to significantly worsen active TB. These data emphasize the potential impact of stringent screening of glycemic responses in TB patients at the time of diagnosis even if diagnostic criteria for diabetes are not met [20], [52]. Our data indicate that inflammation-associated insulin resistance during Mtb infection may be an additional factor contributing to hyperglycemia. Based on our data, impaired control of Mtb infection, amplification of the inflammatory response and exacerbated extrapulmonary dissemination are evident in association with hyperglycemia-mediated AGE accumulation, which may represent a central mechanism in the pathobiology of diabetes-tuberculosis comorbidity.

## Supporting Information

Figure S1 **An oral dose of 400 mg of sucrose induces post-prandial hyperglycemia.** Guinea pigs challenged at time 0 with 400 mg of sucrose orally developed postprandial hyperglycemia of 2.14 fold over baseline that peaked at 45 minutes post-administration. n = 5
(TIF)

Figure S2 **Results of the OGTT revealed no evidence of glucose intolerance consistent with non-diabetic hyperglycemia.** Oral glucose tolerance tests were performed on all four treatment groups at days 30 (A) and 60 (B) of *Mtb* infection. No significant differences are appreciated between treatment groups at any of the 0, 60 or 120 minute time points on either day 30 or day 60 of infection. n = 5
(TIF)

Figure S3 **TB lesion necrosis was not significantly increased by sucrose treatment.** Necrosis within TB lesions was quantified by stereology in lung, lymph node and spleen on both days 30 and 60 of infection and no significant differences were present between sucrose-treated and water-treated guinea pigs. n = 10
(TIF)
