# Supplementary figures and images for: Non-Diabetic Hyperglycemia Exacerbates Disease Severity in Mycobacterium tuberculosis Infected Guinea Pigs
Source: PLoS One. 2012 Oct 4;7(10):e46824. doi: 10.1371/journal.pone.0046824 (PMC3464230; doi:10.1371/journal.pone.0046824)

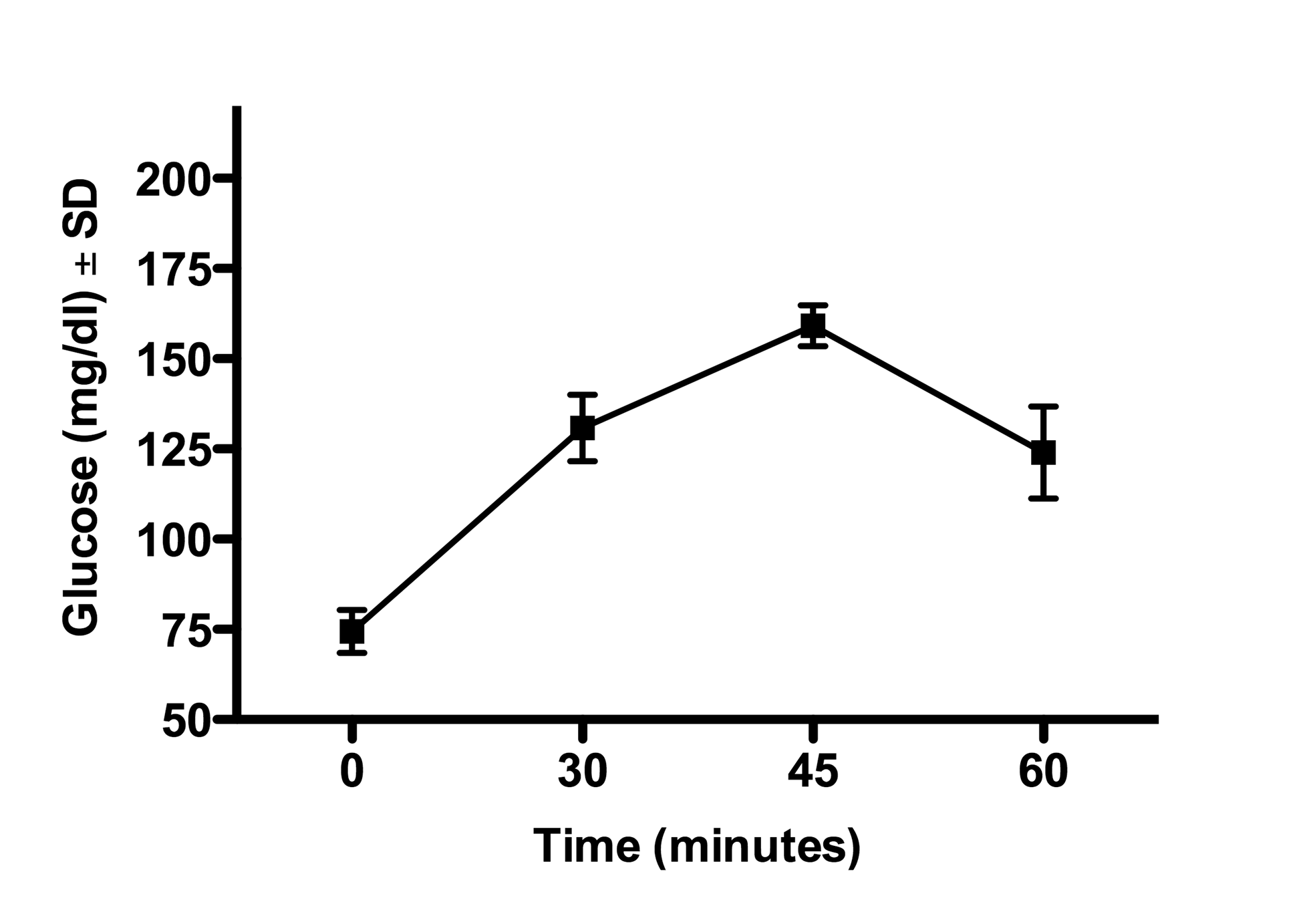

Supplement: Figure S1 — An oral dose of 400 mg of sucrose induces post-prandial hyperglycemia. Guinea pigs challenged at time 0 with 400 mg of sucrose orally developed postprandial hyperglycemia of 2.14 fold over baseline that peaked at 45 minutes post-administration. n = 5 (TIF) [file pone.0046824.s001.tif]

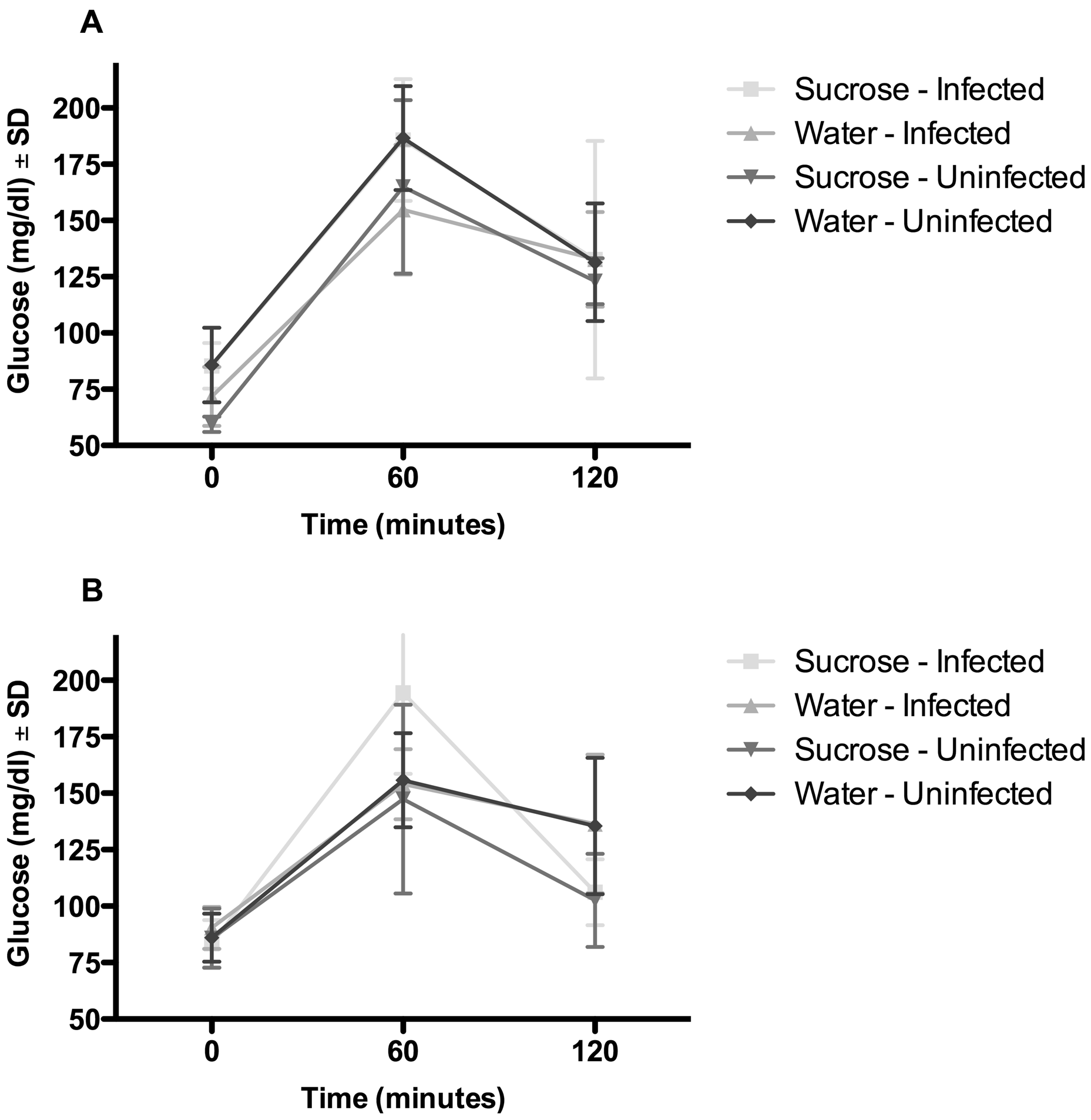

Supplement: Figure S2 — Results of the OGTT revealed no evidence of glucose intolerance consistent with non-diabetic hyperglycemia. Oral glucose tolerance tests were performed on all four treatment groups at days 30 (A) and 60 (B) of Mtb infection. No significant differences are appreciated between treatment groups at any of the 0, 60 or 120 minute time points on either day 30 or day 60 of infection. n = 5 (TIF) [file pone.0046824.s002.tif]

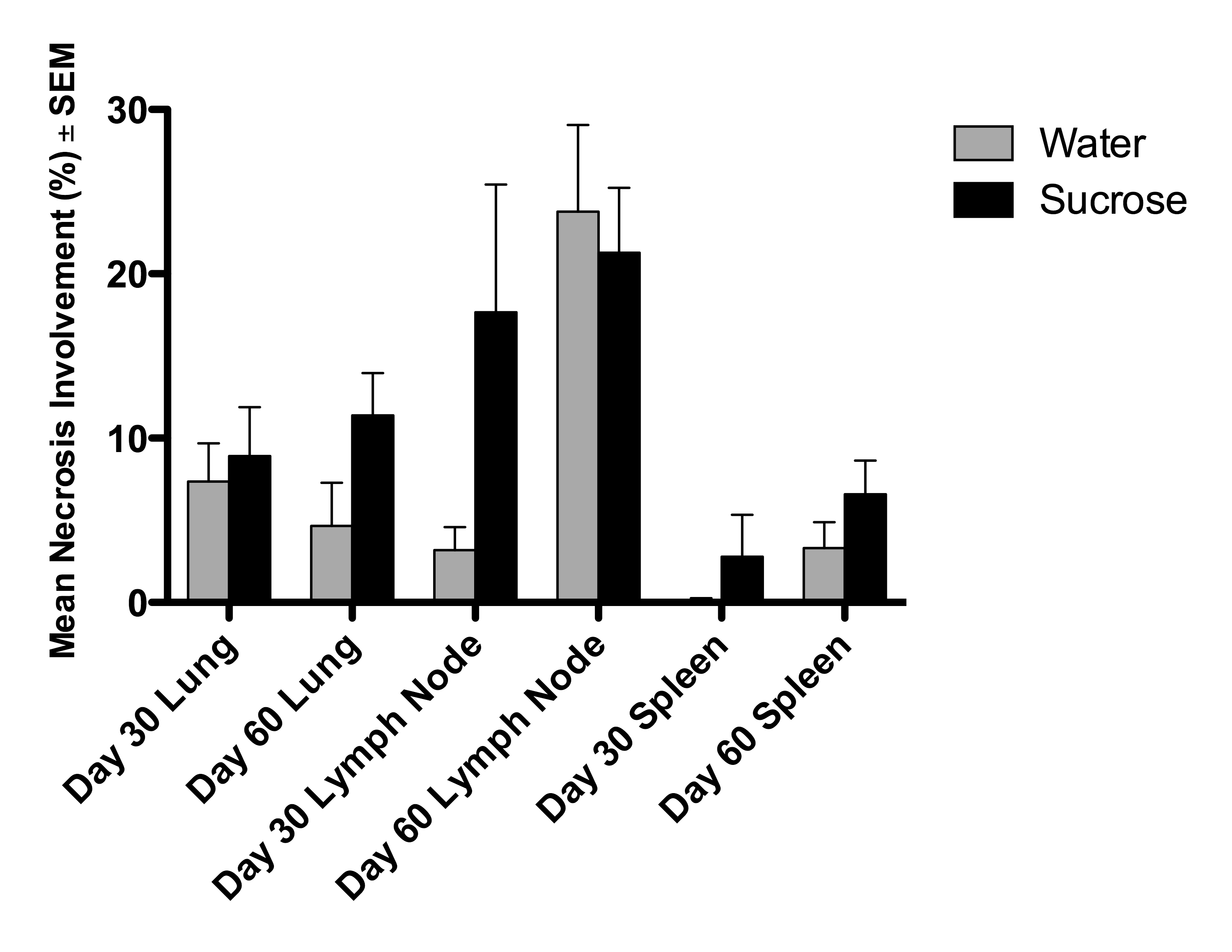

Supplement: Figure S3 — TB lesion necrosis was not significantly increased by sucrose treatment. Necrosis within TB lesions was quantified by stereology in lung, lymph node and spleen on both days 30 and 60 of infection and no significant differences were present between sucrose-treated and water-treated guinea pigs. n = 10 (TIF) [file pone.0046824.s003.tif]
